# Separation of Lipoproteins for Quantitative Analysis of ^14^C-Labeled Lipid-Soluble Compounds by Accelerator Mass Spectrometry

**DOI:** 10.3390/ijms25031856

**Published:** 2024-02-03

**Authors:** Jennifer C. Chuang, Andrew J. Clifford, Seung-Hyun Kim, Janet A. Novotny, Peter B. Kelly, Dirk M. Holstege, Rosemary L. Walzem

**Affiliations:** 1 Nutrilite, 5600 Beach Blvd., Buena Park, CA 90621, USA; jennifer.chuang@amway.com; 2 Department of Nutrition, University of California, Davis, CA 95616, USA; 3 Department of Applied Bioscience, College of Life and Environmental Science, Konkuk University, Seoul 143-701, Republic of Korea; kshkim@konkuk.ac.kr; 4 U.S. Department of Agriculture, Beltsville Human Nutrition Research Center, 10300 Baltimore Avenue, Beltsville, MD 20705, USA; janet.novotny@usda.gov; 5 Department of Chemistry, University of California, Davis, CA 95616, USA; 6 UC Davis Analytical Lab, University of California, Davis, CA 95616, USA; 7 Poultry Science Department, Graduate Faculty of Nutrition, Texas A&M University, College Station, TX 77843, USA

**Keywords:** accelerator mass spectrometry, isopycnic centrifugation, lipoproteins, fluorescent imaging, α-tocopherol tracer analysis

## Abstract

To date, ^14^C tracer studies using accelerator mass spectrometry (AMS) have not yet resolved lipid-soluble analytes into individual lipoprotein density subclasses. The objective of this work was to develop a reliable method for lipoprotein separation and quantitative recovery for biokinetic modeling purposes. The novel method developed provides the means for use of small volumes (10–200 µL) of frozen plasma as a starting material for continuous isopycnic lipoprotein separation within a carbon- and pH-stable analyte matrix, which, following post-separation fraction clean up, created samples suitable for highly accurate ^14^C/^12^C isotope ratio determinations by AMS. Manual aspiration achieved 99.2 ± 0.41% recovery of [5-^14^CH_3_]-(2R, 4′R, 8′R)-α-tocopherol contained within 25 µL plasma recovered in triacylglycerol rich lipoproteins (TRL = Chylomicrons + VLDL), LDL, HDL, and infranatant (INF) from each of 10 different sampling times for one male and one female subject, n = 20 total samples. Small sample volumes of previously frozen plasma and high analyte recoveries make this an attractive method for AMS studies using newer, smaller footprint AMS equipment to develop genuine tracer analyses of lipophilic nutrients or compounds in all human age ranges.

## 1. Introduction

Accelerator mass spectrometry (AMS) is most commonly known for its use to radiocarbon date materials in geochronology, archeology, and anthropology studies via sensitive and highly accurate ^14^C/^12^C isotope ratio determinations [1,2]. We previously identified four features of AMS determinations of ^14^C/^12^C isotope ratios that are highly advantageous in long-term in vivo human ADME (absorption, distribution, metabolism, elimination) and PBPK (physiological-based pharmacokinetic) studies of food components, nutrients, and/or new drugs [3]. Those reasons included a low natural abundance and long half-life of ^14^C, as well as its ready incorporation into many natural/organic molecules or compounds, coupled with AMS’s ability to measure ^14^C in attomole amounts. We and others have taken advantage of ^14^C labeling and AMS determinations in biokinetic studies of vitaminers with the view of using this sensitive analytical approach for the accurate determination of quantitative nutrient requirements through the determination of pool sizes and turnover rates in well-nourished and deficient individuals [3,4,5,6,7].

To date, we and others have used a singular plasma pool in AMS studies of lipid-soluble vitamin biokinetics due to the lack of lipoprotein separation methodologies with sufficient speed and resolution to allow for the frequent sampling needed for the accurate biokinetic modeling of these nutrients within the vascular compartment. This approach is non-optimal. Lipoproteins are spherical particles with a lipid core covered by a layer of phospholipids and specialized proteins. Lipoproteins are a mixed analytic pool that was heterogeneous in lipid content, particle density, and physiological function. Moreover, lipid-soluble analytes can move rapidly among lipoproteins of different density classifications, i.e., chylomicron (CHY), very-low-density lipoprotein (VLDL), low-density lipoproteins (LDLs), and high-density lipoproteins (HDLs) via lipid transfer proteins [8] as well as transport following binding to albumin or other plasma proteins [9,10]. 

Traditional lipoprotein separation methodologies require fresh plasma, which poses a substantial challenge to the long-term detailed biokinetic modeling of lipoprotein-associated molecules which collect frequent and extended blood sampling. Methods to separate lipoproteins from fresh plasma must be performed promptly after blood collection, which can often be logistically difficult, especially after extended blood collections for kinetic experiments. In earlier experiments by our group, sequential density gradient ultracentrifugation (SDGU) methods were used to separate and isolate individual lipoprotein density classes for subsequent ^14^C tracer quantification by AMS [11]. Although the adequate resolution of lipoprotein density classes was achieved by SDGU, tracer recovery was low and inadequate when coupled with AMS due to the unique requirements for this analytical technique [3,12]. Furthermore, the three-day SDGU procedure was cumbersome for analyzing large number of samples generated by biokinetic studies. Due to these limitations, we searched for an alternative to SDGU that provided robust and efficient means to separate lipoproteins compatible with the requirements of AMS. 

Metal ion complexes of EDTA were shown to form solutes usable for continuous density gradient separations of lipoproteins [13]. The subsequent imaging of fluorescently labeled lipoproteins showed that the method was capable of high-resolution separations using microliter volumes of plasma or serum [13]. This technique appeared to be suitable for combination with AMS, which requires the use of small sample volumes with a low carbon content when utilizing ^14^C-labeled tracers [3]. A final density solution comprised of a cesium–bismuth–EDTA complex, CsBiEDTA, was recommended as it formed a highly reproducible continuous density gradient capable of resolving VLDL, LDL, HDL, and infranatant fractions within 6 h [14]. However, use of the original density media and fraction recovery techniques [15,16] produced low and variable recoveries with clinical samples from an AMS study [4], necessitating present studies to optimize its use in conjunction with AMS. Method optimization requires changes to constituent salts of the density medium, pH buffering, lipoprotein imaging, and a density class recovery technique to render the method suitable for use in long-term biokinentic studies of lipid-soluble nutrients. The present experiments used sample material from a long-term study of [5-^14^CH_3_]-(2R, 4′R, 8′R)-α-tocopherol in humans [3] with the aim of developing data for subsequent biokinetic modeling analysis in which lipoprotein subclasses were resolved. The modified procedure is generally applicable to biokinentic studies of lipid-soluble nutrients.

## 2. Results and Discussion

### 2.1. Inadequate Separation of Chylomicrons from VLDL by Water Overlayment

**Figure 1:**
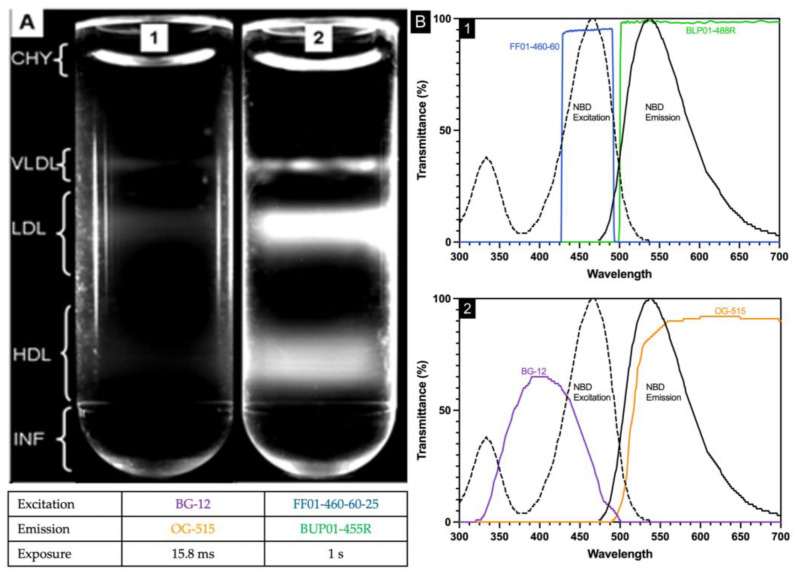
Comparison of optical filters and exposure time to maximize contrast and exposure without total image saturation. Image of 25 μL plasma, 5 μL of NBD C6-Ceramide, and 970 μL of 12% Cs2CdEDTA density solution imaged using either Quik Mod filters according to [13] ((**A1**), filter spectral cut-offs shown in (**B1**)) or Semrock filters described in present study, ((**A2**), filter spectral cut-offs shown in (**B2**)). Note difference in exposure time, 15.8 ms vs. 1 s.

Plasma (25 µL), NBD C_6_-ceramide (5 µL), and CsBiEDTA 15% *w*/*v* (970 µL) were mixed and centrifuged at 120,000 RPM at 4 °C for 6 h. Routine images were taken immediately after an aliquot of water (200 µL) was layered atop the meniscus. Water layering was originally published to prevent meniscus artifact during triglyceride-rich lipoprotein imaging [16]; water layering did not consistently float CHY away from VLDL. In Figure 1, the area bracketed as CHY shows the curved meniscus artifact arising from a light scatter.

### 2.2. Modification of Density-Gradient-Forming Salts

While the originally described CsBiEDTA solution was effective in lipoprotein separation, the use of Cs_2_CO_3_ to adjust pH created a solution where pH increased with storage time, and which added too much carbon for effective AMS analysis. Systematic modifications to the buffer system were undertaken to create a pH-stable and lower-carbon-content buffer.

The starting CsBiEDTA solution was synthesized from cesium carbonate (Cs_2_CO_3_), bismuth carbonate ((BiO)_2_CO_3_), and H_4_EDTA as described [14]. Briefly, 0.4 mol of H_4_EDTA, 0.2 mol of (BiO)_2_CO_3_, and 0.2 mol of Cs_2_CO_3_ were dissolved in 80 mL of water and refluxed for 2 h under high heat and then cooled to room temperature. In the original procedure, solution pH was adjusted to pH 7.40 ± 0.05 with Cs_2_CO_3_, and then the final volume was adjusted to 200 mL. 

Several buffers were evaluated for their ability to maintain a stable pH and cleanly separate lipoprotein classes (Table 1). The buffers TES and HEPES were unable to bring the final density solution to pH 7.4. Use of small amounts of the strong base CsOH to increase the initial density solution pH proved problematic as white precipitants formed within an hour and the pH increased from ~pH 7 to over pH 10. Sodium hydroxide addition to the density solution held pH = 7.4 for a week but disrupted the formation of density gradients formed during centrifugation and caused a non-ideal lipoprotein separation.

Only TRIS and phosphate buffer sustained a physiologic pH for over a week. However, the presence of phosphate ions disrupted the density-gradient-forming properties of CsBiEDTA and resulted in a loss of resolution in the lipoprotein separation. Density solution performance at 5 °C following pH stabilization at 7.4 was best with 0.5 mM TRIS.

### 2.3. Carbon Reduction in Density Gradient Solution

To reduce the buffer’s carbon content, non-carbonate salts of cesium and bismuth were evaluated. Salts tested included cesium acetate, Cs_2_CO_3_, cesium hydroxide, bismuth carbonate, bismuth oxide, Bi_2_O_3_, and bismuth phosphate. Density solutions made with cesium acetate or bismuth phosphate formed crystals overnight and so were discontinued. Only Bi_2_O_3_ with Cs_2_CO_3_ and H4EDTA remained soluble, rendering Bi_2_O_3_ as the optimum alternate reactant. Density solutions formulated with Bi_2_O_3_ had a starting pH of 7.1 instead of 4.5 and so required less buffer salts to achieve a final pH of 7.4. Density solution made with Cs_2_CO_3_, Bi_2_O_3_, and H_4_EDTA, whose pH was adjusted to 7.4 with TRIS, remained stable for over 6 months. These reactants and final pH-stabilized buffer, termed AMS-LP, became the new density medium for further lipoprotein fractionation studies.

The AMS-LP density medium could be reduced to crystals by slow evaporation. These crystals were readily rehydrated for immediate use. Slow evaporation produced larger clear crystals, while fast water removal produced small crystals in the form of a white powder. The clear crystals provided superior dissolution and stability of the re-hydrated density medium compared to the white powder form; the latter did not fully dissolve, even following reflux, and led to an inaccurate density solution concentration.

### 2.4. Imaging Filter Optimization

In the original procedure [13], two optical filters (BG12 and OG515, Edmund Industrial Optics, Barrington, NJ, USA) were used. The BG12 band-pass filter centered at 407 nm with a width of 104 nm at 80% transmittance full-width half-maximum. The OG-515 long pass filter has a 515 ± 6 nm cut-off position and bandpass limit of 580 nm with 80% transmittance. NBD C6-ceramide is weakly fluorescent in water, but its fluorescence increases in aprotic solvents and other nonpolar environments such as lipoproteins with excitation/emission maxima ~466/536 nm and was used as the detector molecule. The spectral properties of NBD C6-ceramide were better matched by the filter combination of FF01-460-60-25 and BLP01-488R-25 as the excitation bandpass filter (FF01-460-60-25) wavelength is centered at 465 nm with a bandwidth of 60 nm, while the emission long pass filter (BLP01-488R-25) allows wavelengths greater than 500 nm to pass through. The new filter combination allowed exposure time to be increased significantly without background saturation, assuring that the entirety of the lipoproteins within each density region were visualized (Figure 1).

### 2.5. Determination of Working Concentration and Volume

**Figure 2:**
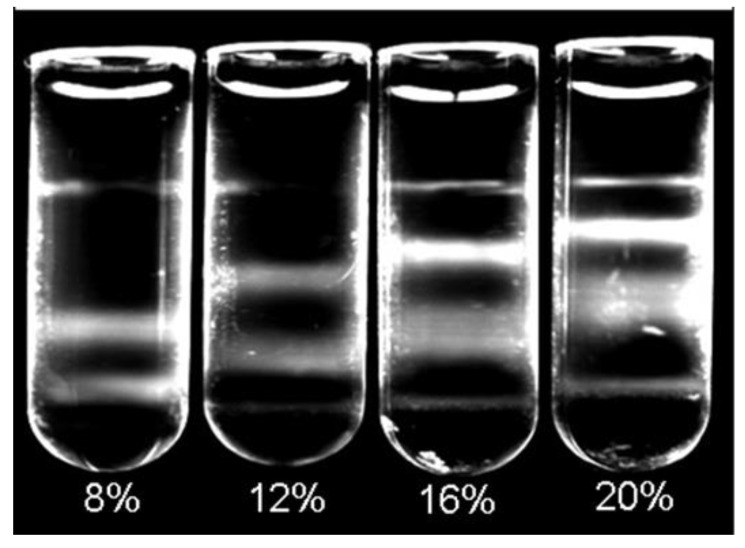
Determination of working concentration and volume of CsBiEDTA solution. CsBiEDTA density solution at 8%, 12%, 16%, and 20% *w*/*v* showing effective separation with 12–16% *w*/*v*.

As different volumes and concentrations of CsBiEDTA were reported for various lipoprotein separations depending upon specific application requirements [13,14], we tested a range of density solution concentrations and volumes to optimize lipoprotein separation for AMS determinations. Dilutions of 20% *w*/*v* of AMS-LP were used to create a set of density solutions ranging from 5 to 20% *w*/*v*. A total of nine different concentrations were evaluated. Each density concentration was evaluated using the combination of 25 µL plasma, 5 µL NBD C6-ceramide, and 970 µL of appropriate AMS-LP dilution. Prepared mixtures were then centrifuged and imaged according to the standard protocol. AMS-LP solutions ranging from 8 to 16% *w*/*v* were able to separate VLDL, LDL, and HDL from the infranatant using the above-described separation protocol. The final flotation positions of CHY and VLDL were not affected by the density solution concentration as these fractions consistently floated proximal to the meniscus due to particle densities ≤ 1.0 g/mL. The LDL and HDL regions were sensitive to changes in density solution concentration (Figure 2).

The volume determinations employed replicate 25 µL plasma aliquots mixed with 5 µL of NBD C6-ceramide. Three aliquots, 970 µL, 1070 µL, and 1170 µL, each of 8% 12%, 14%, and 16% *w*/*v* AMS-LP, were added into a polycarbonate centrifuge tube. Each tube was covered with parafilm, gently vortexed for 15 s, incubated at 5 °C for 30 min, and then centrifuged at 200,000 RPM at 5 °C for 6 h. Samples were imaged immediately after careful overlayment of 200 µL of water. 

While CYL and VLDL consistently located near the meniscus, the final relative position and band-shape of LDL and HDL within the tube varied with volume. As the total volume increased, LDL and HDL bands shifted upward in the tube and the HDL band spread over a larger area, weakening its fluorescence signal. For this reason, 970 µL of density solution at concentrations between 12 and 16% CsBiEDTA was used in conjunction with 25 µL of plasma and 5 µL of NBD fluorophore (total volume of 1000 µL). A 15% solution provided a conveniently calculated dilution.

### 2.6. Density Gradient and Freezing

**Figure 3:**
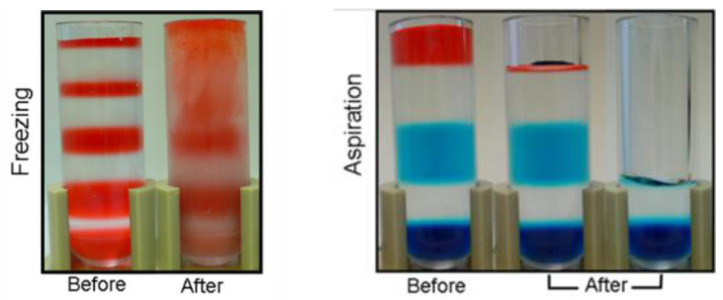
Effect of recovery method on density subclass separation. **Left** panel shows mixing in d < 1.075 g/mL regions of a model density gradient following freeze/cut procedure as per [15]. Freezing: Density range from top to bottom: 1.00 g/mL (red), 1.010 g/mL (clear), 1.025 g/mL (red), 1.050 g/mL (clear), 1.075 g/mL (red), 1.125 g/mL (clear), 1.200 g/mL (red), 1.350 g/mL (clear), 1.400 g/mL (red). **Right** panel shows non-disturbance of density gradient using aspiration, as described in present study. Aspiration: Density range from top to bottom: 1.00 g/mL (red), 1.050 g/mL (clear), 1.125 g/mL (aqua), 1.350 g/mL (clear), 1.400 g/mL (navy).

Tube freezing followed by the excision of density regions by sawing was proposed as a means for complete sample collection for HDL studies [15]. Initial tests of the procedure using AMS-LP suggested that thermal mixing occurred. To test this possibility, the effects of freezing on density gradients were assessed using a model AMS-LP matrix in which red food coloring was used to alternatively dye density regions, and these were then layered manually rather than being formed centrifugally. This model density gradient comprised solutions of the following densities: 1.00 g/mL, 1.010 g/mL, 1.025 g/mL, 1.050 g/mL, 1.075 g/mL, 1.125 g/mL, 1.200 g/mL, 1.350 g/mL, and 1.400 g/mL. The prepared tubes were slowly frozen by holding over liquid nitrogen vapor. Images were taken immediately after the samples were removed from the liquid nitrogen vapor. As shown in the top panel of Figure 3, thermal mixing occurred in fractions with densities < 1.075 g/mL. The addition of additives such as DMSO, ethylene glycol, and sucrose (ranging from 8 to 16%) as modifiers of the water overlayment during the freezing process was ineffective in the prevention of thermal mixing in low-density regions of the gradient during freezing. 

### 2.7. Density Gradient and Aspiration

Model gradients that had not been frozen were aspirated manually to test this alternative means of lipoprotein subfraction collection. A syringe fitted with a 28-gauge 1-inch blunt tip needle (250 µL, Hamilton, Reno, NV, USA) was used to aspirate alternatively dyed layers of the model gradients (Figure 3). This model density gradient comprised solutions of the following densities 1.00 g/mL (red), 1.050 g/mL (clear), 1.125 g/mL (aqua), 1.350 g/mL (clear), and 1.400 g/mL (navy). Aspiration was found to extract density fractions without disrupting subsequent density layers and was more efficient than freezing and cutting. The differently colored layers showed that direct aspiration did not completely remove one density region from another. However, careful overlayment of 200 µL water following density fraction aspiration and aspiration of this 200 µL of water effectively removed the residual.

### 2.8. Density Fraction Desalting Prior to Accelerator Mass Spectrometry (AMS)

Three extraction and desalting methods were evaluated in triplicate using isotope recovery measured by AMS as the criterion [17]. Liquid–liquid extraction treated AMS-LP-separated lipoprotein fractions with 1 mL hexane three times. Hexane was shown to be one of the few common solvents that did not prompt crystal formation in AMS-LP. The three extracts were combined prior to AMS measurement. The second method tested was an ultrafiltration method described for lipoproteins [16] with density fractions filtered through a 100,000 NMWCO centrifugal filter (Millipore, Danvers, MA, USA) to remove AMS-LP prior to thrice washing with 1 mL water. A third, solid-phase extraction method used an Oasis HLB µElution Plate 30 µm (Waters, Milford, MA, USA) cartridge conditioned sequentially with 300 µL each of toluene, isopropanol, methanol, and finally water. After loading each lipoprotein fraction, samples were washed three times with 300 µL of 95/5 water/methanol following the manufacturer’s instructions. Fat-soluble compounds were retained on the sorbent and subsequently eluted with 300 µL of 95/5 hexane/isopropanol. The lipoprotein fractions were analyzed by AMS [3]. As shown in Table 2, sample recovery was greatest and variability lowest using the solid-phase extraction procedure, and this method was adopted for subsequent studies. 

### 2.9. Verification of Improved Method Performance for AMS Studies

Variability in the recovery of ^14^C within individual density fractions was used to validate the optimized procedures. Method linearity was tested in duplicates of 30, 25, 5, 2, and 0.5 µL of identical plasma samples. A linear relationship between ^14^C content and plasma volume was noted for volumes between 0.5 and 30 µL of plasma, Table 3, y = 80.74x − 27.69, R^2^ = 0.983, n = 10. Separation and quantitation carried out with 2–25 µL of plasma produced complete recoveries of [5-^14^CH_3_]-(2R, 4′R, 8′R)-α-tocopherol. Using larger or smaller amounts of plasma with this method, specifically 30 or 0.5 µL, overestimated the amount of [5-^14^CH_3_]-(2R, 4′R, 8′R)-α-tocopherol to 122.8% and 131.7% of the actual amount, respectively (Table 3).

Intra- and inter-day precision was determined using identical 25 µL plasma samples and measured in triplicate on 5 separate days. Table 4 shows that intra-day variability was <4.1%, and the inter-day variability was <6.3%. 

**Figure 4:**
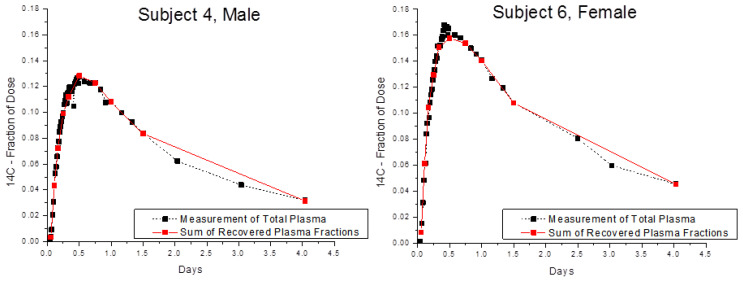
Fraction of ^14^C-α-tocopherol dose over time in total plasma (■) and in the sum of plasma fractions (■). Blood was collected from two volunteers, Subject 4 = male; Subject 6 = female, after consumption of 3.70 kBq of [5-^14^CH_3_]-2R, 4′R, 8′R) -α-tocopherol.

Finally, the optimized method was tested using blood samples collected from Subjects 4 and 6 in a biokinetic study of α-tocopherol [4]. Recovery of ^14^C in whole blood and in blood fractions was compared after two volunteers consumed 3.70 kBq of [5-^14^CH_3_]-(2R, 4′R, 8′R)-α-tocopherol (Figure 4) and provided serial blood samples for 4 days after dosing. The amounts of ^14^C in whole plasma and in the sum of plasma fractions were in very good accord with an average recovery of near 100% for 2, 5, and 25 μL plasma.

## 3. Methods and Materials

### 3.1. Materials

All chemicals were reagent grade or better. In the optimized lipoprotein separation, the CsBi-EDTA-TRIS density gradient material was made from desiccated salts of cesium carbonate (CsCO_3_ 99%, TCI America, Portland, OR, USA), bismuth oxide (Bi_2_O_3_ 98.5%, TCI America, Portland, OR, USA), and H_4_EDTA (Sigma Aldrich, St. Louis, MO, USA). Salts, 13.74 g of H_4_EDTA, 10.95 g Bi_2_O_3_, and 7.66 g CsCO_3_, were dissolved in about 90 mL water and refluxed for 2 h under high heat. The systematic testing of possible buffers, described in the Section 2, concluded that TRIS buffer was most suitable. Consequently, in the analyses of the final sample, the CsBi solution was cooled to 24 °C and the pH was adjusted to 7.4 by the addition of 0.25 mM (~2 mL) TRIS buffer (Trizma base, Sigma Aldrich, St. Louis, MO, USA). The solution was brought to a final volume of 100 mL (30% *w*/*v*), filtered, and stored at 5 °C until it was used. Crystallization of the prepared solution by slow evaporation provided a stable form of readily dissolved density material; freshly prepared solutions were pH-stable for at least one year. For sample analysis, the density solution was made fresh and used within a week of preparation. The pH of the solution was measured using a digital pH meter before each use to ensure the correct pH. 

### 3.2. Plasma Collection

Plasma for this study was collected as part of an Institutional Review Board (IRB)-approved biokinetic AMS study in which 0.78 μg of [5-^14^CH_3_]-(2R, 4′R, 8′R)-α-tocopherol providing 3.7 kBq of ^14^C was the tracer [3]. The recommended daily allowance for tocopherol is 15 mg per day, thus the dose administered qualifies as a microdose https://www.fda.gov/media/107641/download (accessed on 10 March 2020) [10]. Study subjects were healthy non-smokers and provided informed consent that included secondary sample analyses. The samples analyzed were less than 1 year old. Following blood collection and plasma harvest, plasma was aliquoted into three separate cryovials and stored at −80 °C prior to use in order to minimize freeze/thaw cycles. Frozen plasma was thawed (1×) at 5 °C and mixed briefly by vortex prior to withdrawing the 25 µL sample used in lipoprotein separations. Samples frozen for 2 years or less and thawed fewer than 3 times gave comparable separations [18]. The characteristics of the individuals whose recovery outcomes following lipoprotein fractionation are reported here were previously published [3,4]. Briefly, Subject 4: Male, 22 years old, 75 kg, BMI 23, HDL 1.6 mmol/L, LDL 2.8 mmol/L, TG 0.5 mmol/L, plasma alpha-tocopherol 20 micromol/L. Subject 6: Female, 26 years old, 59 kg, BMI 21, HDL 1.5 mmol/L, LDL 3.2 mmol/L, TG 1.5 mmol/L, plasma alpha-tocopherol 17 micromol/L.

### 3.3. Ultracentrifugation and Fluorescent Labeling of Plasma Samples

In its optimized embodiment, lipoprotein density distribution was determined following the incubation of 25 μL plasma with 5 μL NBD C_6_-ceramide (1 mg NBD-C_6_-Ceramide/mL DMSO, Molecular Probes, Inc., Eugene, OR) and 970 μL of a 15% *w*/*v* CsBiEDTA density solution at 5 °C for 30 min. Prepared samples were placed in thick-walled 1.5 mL polycarbonate ultracentrifuge tubes (part # S300535, Hitachi, Tokyo, Japan) prior to insertion into a TLA-120.2 rotor and Optima TLX ultracentrifuge (Beckman Coulter, Brea, CA, USA). Lipoproteins were separated by centrifugation at 120,000 RPM at 5 °C for 6 h. Water (200 µL) was carefully overlaid to remove meniscus parallax, and an image of the ultracentrifuge tube was immediately taken by a digital Microfire camera (Optronics, Goleta, CA, USA) with a Fiber-Lite MH100A Illuminator (Fiber-Lite, Lawrence, CA, USA) as the light source.

### 3.4. Fluorescent Imaging

The tube holder, digital camera, and illuminator were positioned orthogonally to each other on an optical bench. The respective filters (Semrock, Rochester, NY, USA) were chosen to match NBD excitation (465 nm/60 nm bandwidth, part # FF01-460-60-25) and emission (>500 nm/long pass, part # BLP01-488R-25) wavelengths, respectively. Images were captured using PictureFrame [TM] application 3.0 for the MicroFIRE 3.0 (serial number A) camera set to an exposure time of 1.0 s, a gain of 1.0, and a target intensity of 30%. To generate a lipoprotein density profile, the pixel values of the image file were converted into fluorescence intensities by Origin 7.0 (OriginLab, Northampton, MA, USA). 

### 3.5. Density Fraction Recovery

A syringe fitted with a 28-gauge 1-inch blunt tip needle (250 µL, Hamilton, Reno, NV, USA) was used to aspirate density fractions without disrupting subsequent density layers. Because direct aspiration did not completely remove one density region from another, 200 µL water was carefully overlaid following density fraction aspiration, and the aspiration of this 200 µL of water effectively removed the residual amounts of the preceding, less dense, subfraction.

### 3.6. Density Fraction Desalting Prior to Accelerator Mass Spectrometry (AMS)

An Oasis HLB µElution Plate 30 µm (Waters, Milford, MA, USA) was conditioned sequentially with 300 µL each of toluene, isopropanol, methanol, and finally water. Lipoprotein fractions were loaded onto the solid-phase extraction device and washed three times with 300 µL of 95/5 water/methanol following the manufacturer’s instructions. The retained fat-soluble compounds were then eluted with 300 µL of 95/5 hexane/isopropanol. 

### 3.7. Analysis of Total Carbon (TC), ^13^C, and ^14^C

Solid graphite or graphite-like materials were ideal for the ^14^C-AMS because they produced a reliable ion current (C^−^) with minimum sample-to-sample contamination. Plasma and lipoprotein subfractions containing microgram amounts of carbonaceous material were converted to graphite as described [17]. Briefly, samples of interest containing 1 mg of C were first oxidized to CO_2_ prior to a subsequent reduction of CO_2_ to filamentous, fluffy, fuzzy, or firm graphite-like substances to coat 5 ± 0.4 mg of −400-mesh spherical iron powder catalyst optimized by the use of 100 ± 1.3 mg of Zn dust and a reduction temperature of 500 °C for 3 h [17].

Total carbon contents in all prepared samples were measured as previously described [3]. Briefly, although plasma samples were known to contain a constant % of carbon, aliquots of 25 µL of plasma sample were measured to confirm the provision of ~1 mg of carbon without intersubject variations. For this determination, 25 mL of plasma was lyophilized, wrapped in aluminum foil, and analyzed for total carbon at the Division of Agriculture and Natural Resources Laboratory at the University of California Davis. Total carbon contents in all samples were measured using a Europa 20/20 isotope ratio mass spectrometer (IR-MS, Sercon Ltd., Cheshire, UK). 

### 3.8. Safety Considerations

Preparation of the density medium requires reflux, and proper precautions for that operation include adequate ventilation and heat protection. Analysis of human samples requires the implementation of biological safety procedures in accordance with BL2 restrictions as set forth in http://www.cdc.gov/biosafety/publications/index.htm (accessed on 10 March 2020). Such an authorization was obtained for this study. 

## 4. Conclusions

Bioavailability and rates of utilization are key considerations in the development of dietary reference intakes of nutrients [19]. The most accurate predictions of nutrient requirements arise from a detailed understanding of the kinetics of a particular nutrient’s metabolism. Lipoproteins are the major transport vehicles for fat-soluble dietary components. A thorough understanding of the movement of fat-soluble nutrients through blood requires kinetic analysis of labeled forms of nutrients within the separate lipoprotein fractions over time. The method we have developed has been optimized for tracer studies with [5-^14^CH_3_]-(2R, 4′R, 8′R)-α-tocopherol and AMS. The AMS approach has previously been shown to allow true tracer studies to be performed without perturbing the kinetics of the natural biological system, an artifact that occurs when large doses of tracer are used [20]. Fat-soluble nutrients and dietary components that would benefit from the application of the lipoprotein density fraction method optimized here for use with AMS beyond vitamin E include vitamin D, vitamin K, β-carotene/vitamin A, lycopene, lutein, and other carotenoids. 

The developed method has been optimized for use in AMS biokinetic tracer analyses of lipophilic analytes carried in lipoprotein particles. In this specific embodiment, we have optimized conditions for α-tocopherol analyses. The novel TRIS stabilized, low-carbon, cesium–bismuth–EDTA density solution developed here meets the AMS carbon requirements while forming highly reproducible, pH-stable density gradients. This density medium has simple sample preparation requirements and achieves the baseline separation of lipoprotein density classes within 6 h. The imaging technique used here generated the strongest signals at congregations of most lipoproteins within a density interval. Just as in Espinosa et al.’s study [21], we found that longer exposure time provided more details of the lipoprotein profiles in the regions with low lipoprotein concentrations. However, our changes to optical filters in the originally described imaging procedure reduced background response to light and so allowed for longer sample exposure times that facilitated the visualization of rare lipoproteins at the “edges” of lipoprotein density classes. Improvements to lipoprotein density class recovery and subsequent clean up prior to AMS analysis created an analytical process suitable for the analysis of lipoprotein subclasses from the large number of samples generated in biokinetic studies. Lipoprotein density classes were defined for human samples 60 years ago [22]. Our ability to unequivocally determine that the baseline resolution of individual plasma lipoprotein classes was achieved in each sample will add confidence to subsequent kinetic modeling efforts. One caveat in this regard is the separation of CHY and VLDL, which cannot be definitive with the method described here as it is reliant upon particle floatation properties which can be identical for both intestinally assembled CHYs and liver-assembled VLDL [23,24]. Future refinements to the methodology could include prior CHY separation from VLDL via immunoprecipititation [25].

The novel observation of the thermal mixing of lipoproteins due to the redistribution of gradient material within the d < 1.075 g/mL region with freezing provides an explanation for the previously observed variation in subfraction recovery that prompted these studies. Aspiration of lipoprotein subfractions proved superior as a method of lipoprotein density interval collection in studies where CHY or VLDL subfractions are sought. Thus, while freezing was tractable for the collection of HDL samples (d > 1.075 g/mL) [15] using the originally described density medium for non-AMS applications, it is not useful for applications that require the collection of triglyceride-rich CHY and VLDL separate from LDL as these density regions were mixed thoroughly. 

The optimized separation and recovery methodologies described here exhibited an order of magnitude improvement relative to the direct application of previous methodologies [13,14,15,16] in AMS sample preparation. Similarly, reproducibility was high with day-to-day variation less than 7% and analyte recovery of 100% within the range of 2–25 µL plasma. These analytical qualities render this method sensitive and reliable with ease of data generation that enables the superior examination of individual variability in nutrient metabolism and allows for the reliable monitoring of lipoprotein-related kinetics of analytes in response to environmental variables, e.g., diet or exercise. 

This methodology is likely to enable the address of several long-standing as well as newly emerging areas of lipoprotein biology in relation to the absorption and utilization of lipophilic nutrients or other bioactive molecules. AMS has been slow to be adopted for ADME and PBPK studies due to the large and expensive instrumentation required. However, new equipment with smaller footprints became available by 2007 [2] and proved to be capable of supporting biomedical studies [26,27,28].

## Figures and Tables

**Table 1:** Effects of buffer salt addition to CsBiEDTA density gradient medium ^1^ salt was proved suitable for density solution pH adjustment, see tabular comments for specifics.

Buffer Salt Addition	Observation
Cesium Carbonate	Stable pH for less than 1 week. Continuous increase in pH to >9.0
Phosphate (pka-2 = 7.20)	Stable pH for over 1 week. Disrupted density gradient. Poor lipoprotein separation.
TES	pH < 7.4
HEPES	pH < 7.4
TRIS	Stable pH > 1 year. Lipoproteins well separated.
Cesium Hydroxide	White metal hydride complex formed, rapid pH increase. Poor lipoprotein separation.
Sodium Hydroxide	White metal hydride complex formed, pH stable for >7 days. Poor lipoprotein separation.

^1^ Density solution was made with cesium carbonate, bismuth carbonate, H_4_EDTA. Initial pH of the density solution was 4.53. Buffer salts were used to minimize volume changes.

**Table 2:** Summary of ^14^C recovered (amol) by liquid-liquid, ultrafiltration, and solid phase extraction ^1^.

	Liquid Liquid Extraction	Ultrafiltration	Solid Phase Extraction
Solvent	Hexane	Water	95/5 Toluene /Isopropanol
CHY	15	20	609
VLDL	248	325	1032
LDL	339	321	1317
HDL	152	799	1536
Infranatant ^2^	70	71	476
Sum	824	1535	4970
Recovery, %	18	33	107
CV, %	40	60	5

^1^ Values are the means of triplicate determinations. ^2^ Contains plasma components such as proteins of density > 1.24 g/mL.

**Table 3:** Recovery of ^14^C-α-tocopherol from lipoprotein fractions arising from 0.5–30 µL of plasma.

Neat Plasma, amol ^14^C-α-TOC	Volume of Plasma, μL	Sum of Fractions, amol ^14^C-α-TOC	Recovery %
2101.64	30	2581.31 ± 21.1	122.8%
1751.37	25	1766.32 ± 0.5	100.9%
350.27	5	367.16 ± 0.2	104.8%
140.11	2	146.38 ± 1.6	104.5%
35.03	0.5	46.13 ± 0.19	131.7%

n = 2 for each determination.

**Table 4:** Intra- and inter-day variability (CV, %) of ^14^C (amol) in 25 µL of plasma measured in triplicate over 5 days.

	Intra-Day Variability, % *N* = 3	Inter-Day Variability, % *N* = 15
CHYLO	2.9 ± 0.02	3.9 ^1^ ± 0.01
VLDL	3.5 ± 0.09	6.3 ± 0.02
LDL	3.9 ± 0.08	7.0 ± 0.07
HDL	5.3 ± 0.07	3.9 ± 0.04
Infranatant	4.1 ± 0.04	3.5 ± 0.04

^1^ n = 14 for chylomicra only.

## Data Availability

Data is contained within the article.
